# Dr. Herbert J. Spencer

**Published:** 1915-07

**Authors:** 


### OBITUARY

**Readers are requested to report promptly the death of all physicians in Western New York, or former residents of this region, or graduates of any medical school in Western New York, and to notify the families of the deceased of our desire to publish adequate obituary notices.**

---

**Dr. Herbert J. Spencer,** N. Y. Homoeopathic 1870, died at his home in West Winfield, May 29, aged 68.

